# Not only lymphadenopathy: case of chest lymphangitis assessed with MRI after COVID 19 vaccine

**DOI:** 10.1186/s13027-022-00419-1

**Published:** 2022-03-17

**Authors:** Vincenza Granata, Roberta Fusco, Paolo Vallone, Sergio Venanzio Setola, Carmine Picone, Francesca Grassi, Renato Patrone, Andrea Belli, Francesco Izzo, Antonella Petrillo

**Affiliations:** 1 Division of Radiology, “Istituto Nazionale Tumori IRCCS Fondazione Pascale – IRCCS di Napoli”, Naples, Italy; 2 Medical Oncology Division, Igea SpA, Naples, Italy; 3 Italian Society of Medical and Interventional Radiology (SIRM), SIRM Foundation, Via della Signora 2, 20122 Milan, Italy; 4 Division of Radiology, “Università degli Studi della Campania Luigi Vanvitelli”, Naples, Italy; 5 Division of Hepatobiliary Surgical Oncology, “Istituto Nazionale Tumori IRCCS Fondazione Pascale – IRCCS di Napoli”, Naples, Italy

**Keywords:** Chest lymphangitis, COVID 19, Vaccination, MRI

## Abstract

**Background:**

To date, no paper reports cases of lymphangitis after COVID 19 vaccination. We present a case of lymphangitis after vaccination from COVID 19, in a patient with colorectal liver metastases.

**Methods:**

We described the case of a 56-year-old woman with history of a surgical resection of colorectal cancer and liver metastases, without any kind of drug therapy for about a month. In addition, a recent administration (2 days ago) of Spikevax (mRNA-1273, Moderna vaccine), as a booster dose, on the right arm was reported.

**Results:**

The magnetic resonance (MR) examination showed the effects of the previous surgical resection and five new hepatic metastases, located in the VIII, VI, V, IV and II hepatic segments. As an accessory finding the presence of lymphadenopathy in the axillary area and lymphangitis of the right breast and chest were identified. The computed tomography scan performed a week earlier, and re-evaluated in light of the MR data, did not identify the presence of lymphadenopathy in the axillary area and lymphangitis signs.

**Conclusions:**

Lymphangitis could occur after COVID 19 vaccine and it is important to know this data to avoid alarmism in patients and clinicians and economic waste linked to the execution of various radiological investigations for the search for a tumour that probably does not exist.

*Trial registration*: Not applicable.

## Introduction

A new coronavirus (severe acute respiratory syndrome coronavirus 2, SARS-CoV-2) is the pathogen responsible for the SARS-CoV-2 disease (COVID-19), which has spread throughout the world since December 2019 [1–8]. COVID-19 was defined as a pandemic by the World Health Organization on 11 March 2020. The clinical spectrum of COVID-19 range from flu-like symptoms to respiratory failure, the management of which demands advanced respiratory assistance and artificial ventilation [9–21]. Currently, a valuable therapy has not yet been improved so that mechanical respiratory support is the only treatment in critically ill patients. In this scenario, it has been critical to develop a vaccine as soon as possible, to prevent SARS-CoV-2 infection in order to protect persons who are at high risk for complications. Globally, at the time of writing, 5 January 2022, there have been 290,959,019 confirmed cases of COVID-19, including 5,446,753 deaths, reported to WHO. As of 3 January 2022, a total of 8,693,832,171 vaccine doses have been administered. In Italy, from 3 January 2020 to 6:18 pm CET, 4 January 2022, there have been 6,396,110 confirmed cases of COVID-19 with 137,786 deaths, reported to WHO. As of 19 December 2021, a total of 104,968,360 vaccine doses have been administered [22].

The Italian authorities have used the following vaccination strategy: two vaccine doses, with a booster dose 5–6 months after the end of the vaccination cycle, in patients who have not been infected. A strategy for monitoring vaccines adverse events is based on the collaboration of local and national health structures, assisted by Italian Medicines Agency (AIFA) [23–25].

Since the beginning of their administration, various reactions and adverse effects of the COVID-19 vaccines have been reported and shared world-wide [4, 24–38]. While vaccine-related lymphadenopathy (LAP) with other vaccines is rare, reports of regional LAP in COVID-19 vaccine recipients are gradually increasing along with the rollout of mass COVID-19 vaccination across the world [24]. LAP can be alarming especially in patients with a history of cancer.

To date, no paper reports cases of lymphangitis after COVID 19 vaccination. We present a case of lymphangitis after vaccination from Covid-19, in a patient with colorectal liver metastases.

## Methods

The study was managed according to the Declaration of Helsinki guidelines. Approval by the Institutional Review Board was not needed considering the nature of the study: the description of a single case report. Informed consent was obtained by the patient. On 5 January 2022, a 56-year-old woman patient arrived at Division of Radiology, “Istituto Nazionale Tumori IRCCS Fondazione Pascale – IRCCS di Napoli”, Naples, Italy, to be submitted to liver MRI in the pre surgical setting. In anamnesis, colorectal cancer surgical resection and liver metastases resection were reported, without any kind of drug therapy for about a month. In addition, a recent administration (2 days ago) of Spikevax (mRNA-1273, Moderna vaccine), as a booster dose on the right arm was reported.

MR examination was performed in a pre-surgical setting, in order to identify all new liver metastases with a 1.5 T MR scanner, Magnetom Symphony (Siemens, Erlangen, Germany) equipped with an 8-element body and phased array coils. The MRI study included pre- and post-contrast images obtained before and post intravenous (IV) contrast agent (CA) injection. MRI sequences were coronal Trufisp T2-weighted free breathing; axial Half-Fourier Acquisition Single-Shot Turbo Spin-Echo (HASTE) T2-weighted, with controlled respiration, without and with fat-suppressed (FS) gradient-echo pulse; coronal HASTE T2-weighted, without FS; axial flash in–out phase T1-weighted, with controlled respiration; Volumetric Interpolated Breath-hold Examination (VIBE) T1-weighted SPAIR with controlled respiration before and after the administration of a liver-specific CA, the Gd-EOB-BPTA (Primovist, Bayer Schering Pharma, Berlin, Germany). The patient received 0.1 mL/kg of Gd-EOB-BPTA by means of a power injector (Spectris Solaris® EP MR, MEDRAD Inc., Indianola, IA, USA) at an infusion rate of 2 mL/s.

## Results

**Fig. 1:**
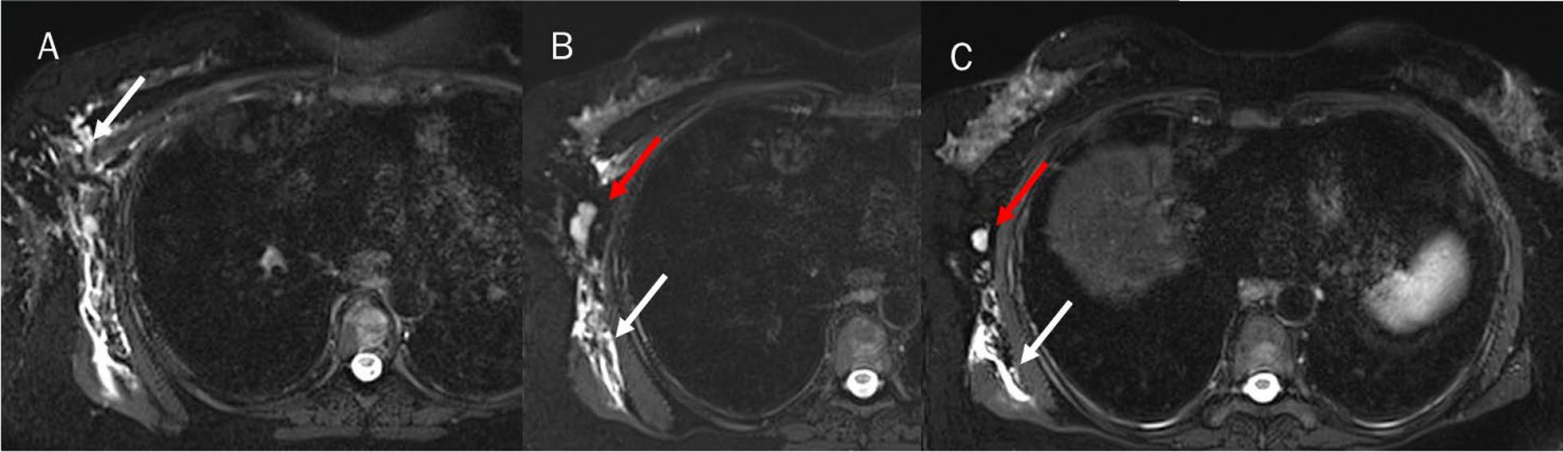
Magnetic resonance study: T2-W fat suppressed sequences in axial plane. White arrows show breast and chest lymphangitis (**A**–**C**). Red arrows show lymphadenopathy (**A**–**C**)

**Fig. 2:**
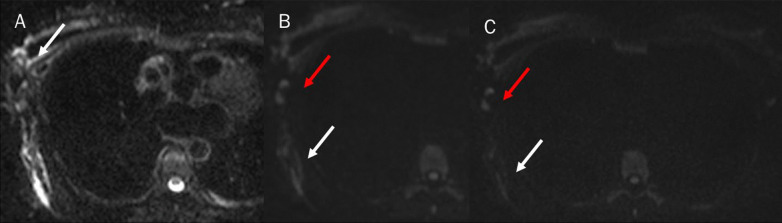
Magnetic resonance study: diffusion weighted imaging (DWI) sequences in axial plane (**A**: b50 s/mm^2^; **B**: b500 s/mm^2^ and **C**: b800 s/mm^2^). White arrows show breast and chest lymphangitis (**A**–**C**). Red arrows show lymphadenopathy (**A**–**C**)

**Fig. 3:**
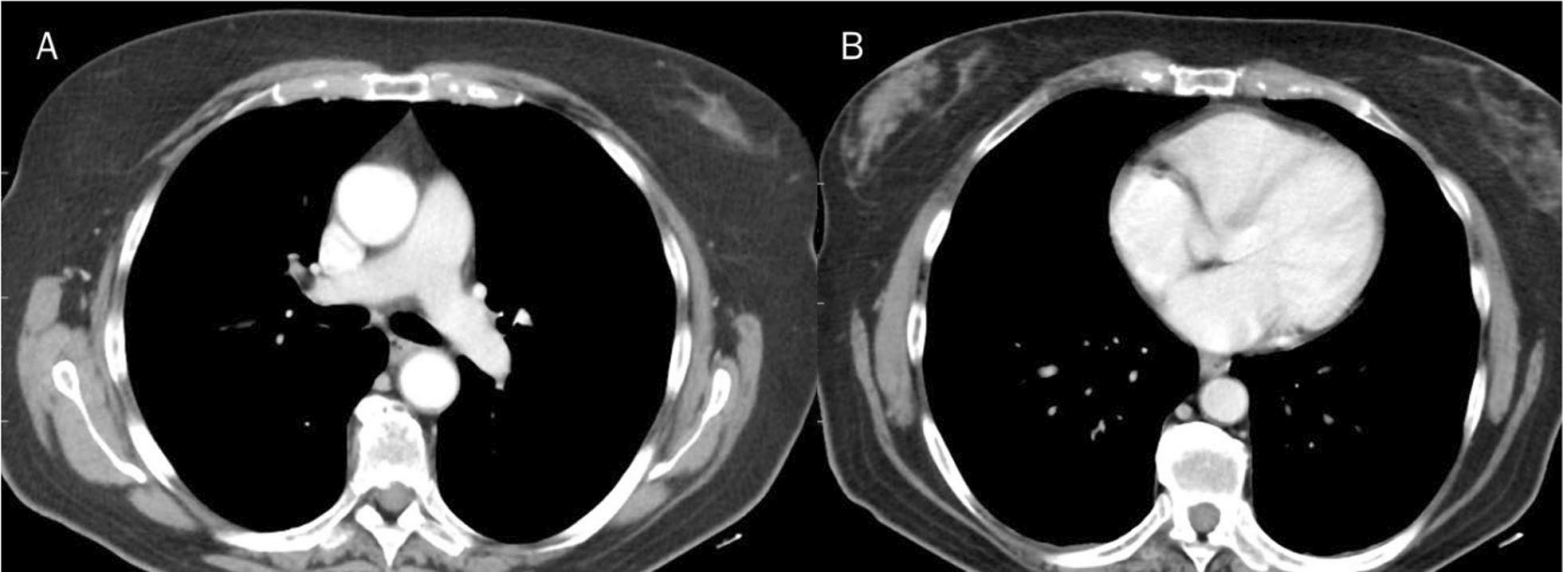
Computed tomography study do not identify the presence of lymphadenopathy in the axillary area and lymphangitis (**A**, **B**)

The liver MR study showed previous surgical resection effects and five new hepatic lesions, located in the VIII, VI, V, IV and II hepatic segments. As accessory findings, the presence of lymphadenopathy in the axillary area and lymphangitis of the right breast and chest were identified (Figs. 1, 2). The competed tomography (CT) scan performed a week earlier, and re-evaluated in light of the MR data, did not identify the presence of lymphadenopathy in the axillary area and lymphangitis (Fig. 3).

Therefore, in the same day, the patient was evaluated with an ultrasound (US) examination, performed by dedicated radiologist, using RS85 Samsung System (Samsung Healthcare GmbH, Schwalbach, Germany) in combination with a linear 5 to 12-MHz array transducer. Four nodes were assessed; the largest diameter was 18 mm with a range from 8 to 18 mm (median value = 12 mm). In the same patient we found different US findings: eccentric cortical thickening with wide echogenic hilum and oval shape, asymmetric eccentric cortical thickening with wide echogenic hilum and oval shape, hypoechoic lymph node round shape without hilum. No anomaly was found on the Doppler study. The US showed skin thickening of the right breast without parenchymal lesions.

The analysis of the patient's radiological archive, allowed to re-evaluate the CT scan performed a week earlier, which showed no other neoplasms other than liver metastases, so as the mammography, performed 2 months earlier, which did not show breast cancer.

Considering the radiological data and the temporal consequentiality with the COVID 19 vaccine, we did diagnosis of lymphangitis due to COVID 19 vaccine.

## Discussion

Although the presence of lymphadenopathy at the site of inoculation of the COVID 19 vaccine is well known [39–42], we have presented, to the best of our knowledge, the first case of cutaneous lymphangitis, in the chest and in the breast due to the COVID 19 vaccine. Knowing this clinical data, which can be found accidentally during radiological examinations, is extremely important, especially in cancer patients. In fact, it is known that various tumours can present with lymphangitis [43–45]. Cutaneous lymphangitis is a rare condition accounting for less than 5% of skin metastases. A literature review identified eight other cases of cutaneous lymphangitis in patients with lung cancer [45], while this condition is present after breast carcinoma in about 23.9% of patients [43], often involving the chest and abdomen and manifesting on average 5 years after surgical removal of the first malignancy. Cutaneous Metastases of breast cancer are usually solitary or multiple nodular pinkish lesions (ranging between 1 and 3 cm). However, several clinical features have been reported in the literature, including telangiectasic carcinoma, erythema-like, erythema annulare centrifugum-like, morphea-like, erysipelas-like, dermatofibroma-like, herpes-zoster-like, and alopecia-like lesions [43].

Skin metastases have the following distribution in women with primary malignancies: breast (69%), large intestine (9%), melanoma (5%), lung (4%), ovary (4%), sarcoma (2%), pancreas (2%), and uterine cervix (2%) [46], and in some cases, it could be the first sign of disease. Therefore, it is our opinion that it is important to spread the knowledge of this new effect of the COVID 19 vaccine for several reasons. Primarily in order not to create alarmism in patients and clinicians and secondly, but no less important for clear economic effects. In fact, a datum seen as an occult sign of neoplasm could determine an economic waste linked to the execution of various radiological investigations, even very expensive, for the search for a tumour that probably does not exist. Radiological examinations that often involve the use of ionizing radiation, with known effects on the health of citizens [47, 48], should be optimize ensuring the lowest reasonably achievable exposure levels (ALARA principle) [49–55].

COVID-19 vaccination-induced lymphadenopathy is increasingly seen on breast imaging; management can be confounded by current or past cancer history. Management of vaccine-related adenopathy detected on breast MRI or other cross-sectional imaging currently varies across radiology practices [56–68]. The management algorithm for adenopathy seen after recent COVID-19 vaccination in a high risk or known breast cancer patient might differ from an average risk patient with adenopathy seen on an otherwise benign-appearing screening mammogram. As the population continues to be vaccinated in larger numbers, adenopathy caused by COVID-19 vaccination will be increasingly seen by breast radiologists and could result in screening call-backs, additional workups, and false positive biopsies [56]. Management of adenopathy in patients with a history of cancer should consider the probability of nodal metastasis, based on original cancer diagnosis (type and location of primary malignancy, expected recurrence patterns) and presence of other suspicious imaging findings or suspicious clinical findings [56]. More recent literature reveals that some centers consider recent COVID-19 vaccination a known inflammatory cause of unilateral axillary adenopathy and therefore recommend a benign assessment (BI-RADS category 2), if adenopathy is ipsilateral to the site of recent COVID-19 vaccination and not palpable. Management recommendations vary by institution, although recommendations have been recently published that offer practical algorithms for management of adenopathy seen after COVID-19 vaccination [57].

Despite the fact that the preventive efficacy of COVID-19 vaccines is debated in clinical trials, the knowledge about what happens following vaccination in the real world is still modest, especially among the general population. Thus, knowing what to expect after vaccination will help with public education, dispelling myths, and lowering the apprehension about COVID-19 vaccines.

## Conclusions

Lymphangitis could occur after COVID 19 vaccine and it is important to know this data to avoid alarmism in patients and clinicians and economic waste linked to the execution of various radiological investigations for the search for a tumour that probably does not exist.

## Data Availability

Data are available at https://zenodo.org/record/6111641#.Yh-hfOjMK3A.
